# Uniform Fe_3_O_4_/Gd_2_O_3_-DHCA nanocubes for dual-mode magnetic resonance imaging

**DOI:** 10.3762/bjnano.11.84

**Published:** 2020-07-08

**Authors:** Miao Qin, Yueyou Peng, Mengjie Xu, Hui Yan, Yizhu Cheng, Xiumei Zhang, Di Huang, Weiyi Chen, Yanfeng Meng

**Affiliations:** 1 Research Center for Nano-Biomaterials & Regenerative Medicine, Department of Biomedical Engineering, College of Biomedical Engineering, Taiyuan University of Technology, Taiyuan, Shanxi 030024, China; 2 Department of MRI, Taiyuan Central Hospital of Shanxi Medical University, Taiyuan, Shanxi 030009, China; 3 Institute of Biomedical Engineering, Shanxi Key Laboratory of Materials Strength & Structural Impact, Taiyuan University of Technology, Taiyuan, Shanxi 030024, China

**Keywords:** 3,4-dihydroxyhydrocinnamic acid (DHCA), dual-mode imaging, Fe_3_O_4_/Gd_2_O_3_-DHCA nanocubes, gadolinium oxide (Gd_2_O_3_), iron(II,III) oxide (Fe_3_O_4_), magnetic resonance imaging (MRI)

## Abstract

The multimodal magnetic resonance imaging (MRI) technique has been extensively studied over the past few years since it offers complementary information that can increase diagnostic accuracy. Simple methods to synthesize contrast agents are necessary for the development of multimodal MRI. Herein, uniformly distributed Fe_3_O_4_/Gd_2_O_3_ nanocubes for *T*_1_–*T*_2_ dual-mode MRI contrast agents were successfully designed and synthesized. In order to increase hydrophilicity and biocompatibility, the nanocubes were coated with nontoxic 3,4-dihydroxyhydrocinnamic acid (DHCA). The results show that iron (Fe) and gadolinium (Gd) were homogeneously distributed throughout the Fe_3_O_4_/Gd_2_O_3_-DHCA (FGDA) nanocubes. Relaxation time analysis was performed on the images obtained from the 3.0 T scanner. The results demonstrated that *r*_1_ and *r*_2_ maximum values were 67.57 ± 6.2 and 24.2 ± 1.46 mM^−1^·s^−1^, respectively. In vivo *T*_1_- and *T*_2_-weighted images showed that FGDA nanocubes act as a dual-mode contrast agent enhancing MRI quality. Overall, these experimental results suggest that the FGDA nanocubes are interesting tools that can be used to increase MRI quality, enabling accurate clinical diagnostics.

## Introduction

Magnetic resonance imaging (MRI) is a noninvasive technique that has been broadly used in the clinical field to assist in disease diagnostics due to its high spatial resolution and capability to differentiate between healthy and unhealthy soft tissues [1–5]. However, due to the substantial increase in the number and complexity of diseases, and also to the fact that MRI has low sensitivity in the absence of contrast, many researchers have been developing strategies to synthesize contrast agents [6–9]. MRI contrast agents can interact with the hydrogen protons that are present in the surrounding tissue, shortening their relaxation times and leading to signal changes [10]. Generally, contrast agents can be divided into two categories depending on the effect they have on MRI. *T*_1_ contrast agents shorten longitudinal relaxation times, generating bright signals [11–15], whereas *T*_2_ contrast agents shorten transverse relaxation times, generating dark signals [16–17]. Even though *T*_1_ contrast agents, such as Gd-DTPA (gadolinium diethylenetriaminepentacetate), are highly advantageous for diagnostic imaging, since they generate bright images, the renal toxicity of Gd-based contrast agents should not be ignored [18–19]. On the other hand, *T*_2_ contrast agents, such as Fe_3_O_4_ nanoparticles, have a lower toxicity in comparison to *T*_1_ contrast agents but might cause artifacts that can disturb anatomical imaging and affect diagnostic accuracy. Since *T*_1_ and *T*_2_ contrast agents pose challenges when used alone in MRI, researchers are starting to focus their efforts on developing strategies to combine *T*_1_ and *T*_2_ into multimodal contrast agents in order to synergistically maximize their advantages while compensating for their drawbacks [20–21]. In addition, this strategy also allows for complementary diagnostic information, which can improve the sensitivity and reliability in detecting lesions.

Given that Fe_3_O_4_ nanoparticles have been extensively investigated as MRI contrast agents due to their biocompatibility, many recent studies concerning *T*_1_–*T*_2_ dual-mode contrast agents use Fe_3_O_4_ nanoparticles [22]. For example, Zhou et al. [23] demonstrated that uniformly distributed Gd-embedded Fe_3_O_4_ nanoparticles significantly enhance the MRI quality since they are *T*_1_–*T*_2_ dual-mode contrast agents.

In contrast, other studies have suggested that nanocubes give better MRI contrast when compared to nanoparticles. This happens because the cubic shape irreversibly introduces a phase difference in *T*_2_ sequence and a better *T*_1_ MR imaging [24–26]. In the present study, the fabrication of novel nanocubes with uniformly distributed Fe_3_O_4_/Gd_2_O_3_ and better MRI contrast was reported. These novel nanocubes were coated with 3,4-dihydroxyhydrocinnamic acid (DHCA), which has a higher exchange efficiency and a lower toxicity in comparison to other modifiers. Moreover, it increases water solubility, which can increase the stability of the nanocubes during in vivo applications [27–28]. In vitro and in vivo tests demonstrated the nontoxicity of the DHCA-coated nanocubes. MRI was performed in vivo, and it showed that the nanocubes increased the imaging contrast and quality, reinforcing the potential that these probes have to be used as an important MRI diagnostic tool.

## Results and Discussion

### Synthesis and characterization of Fe_3_O_4_/Gd_2_O_3_-DHCA (FGDA) nanocubes

**Figure 1:**
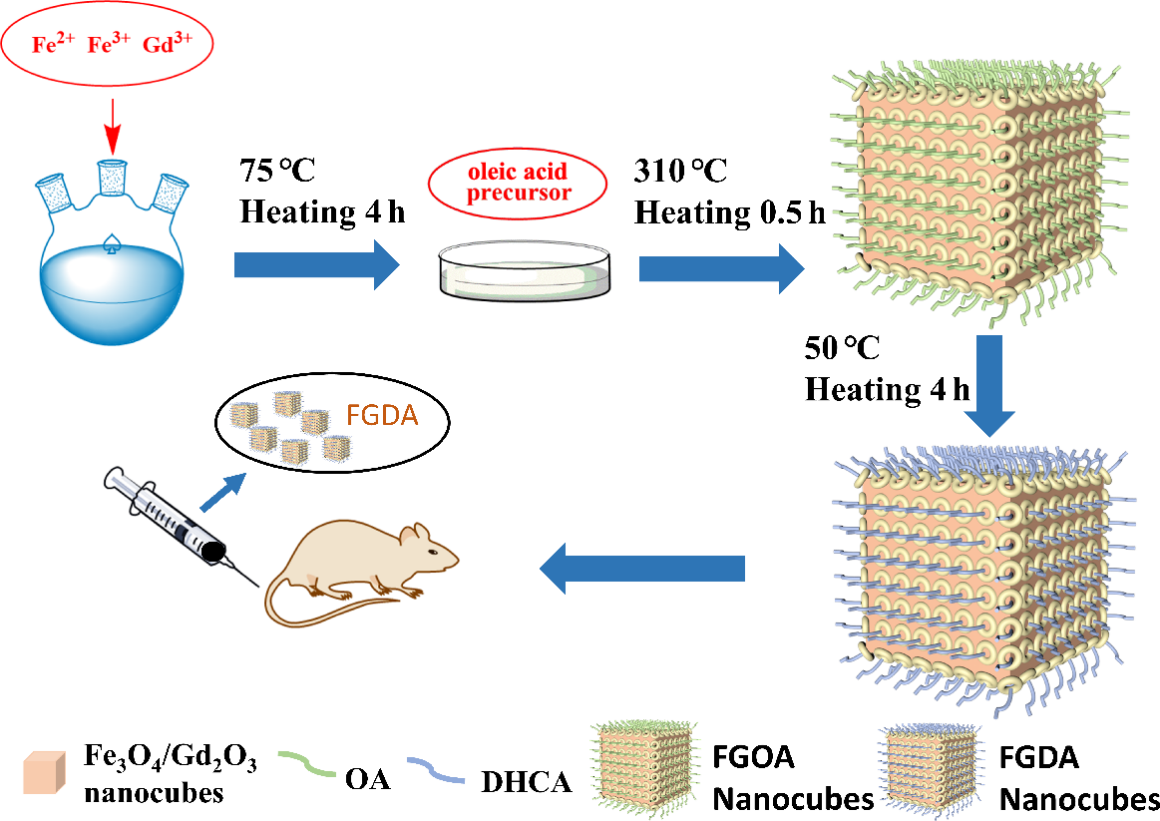
Schematic illustration of FGDA nanocube synthesis. Briefly, a mixture containing sodium oleate, water, ethanol and metal salts was added to a three-necked flask where it was heated and refluxed at 75 °C for 4 h. After cooling, the organic phase obtained was rinsed and dried, forming the metal oleic acid precursor. Next, the precursors were mixed with octadecene and the reaction was heated up to 310 °C and refluxed for 30 min. When the mixture cooled down to room temperature, ethanol was added to precipitate the FGOA nanocubes, which were pooled by centrifugation. After a few rinsing steps, FGOA nanocubes were added to a flask together with DHCA and THF and the mixture was heated up to 50 °C and refluxed for 4 h. After cooling down, NaOH was added to the reaction to precipitate the FGDA nanocubes, which were pooled by centrifugation and resuspended in ultrapure water to be used in in vivo MRI experiments.

**Figure 2:**
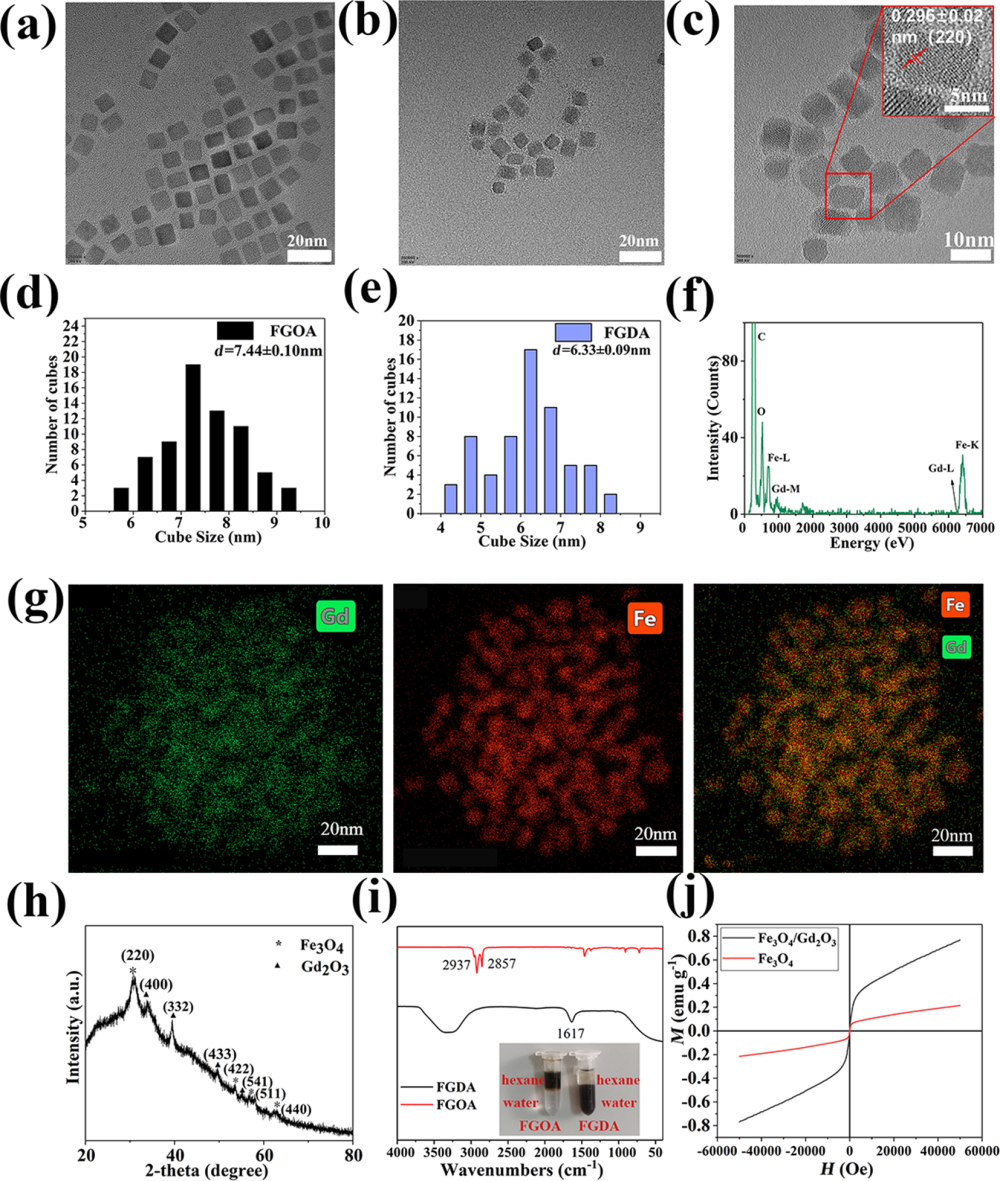
TEM and HRTEM images of FGOA (a) and FGDA (b, c) nanocubes with their respective size distributions (d, e). (f) The EDS spectrum shows the main elements that constitute the FGDA nanocubes. (g) The EDS mapping shows that Gd (green, left panel) and Fe (red, middle) are homogeneously distributed throughout the nanocubes (overlay, right panel). (h) The XRD pattern of FGDA nanocubes shows that the spectrum peaks coincide with the peaks of pure Fe_3_O_4_ (asterisks) and Gd_2_O_3_ (triangles). (i) FTIR spectra of FGOA (red line) and FGDA (black line) nanocubes. (j) Field-dependent magnetization curves (*M*–*H*) of Fe_3_O_4_/Gd_2_O_3_ (black line) and Fe_3_O_4_ (red line) measured using a physical property measurement system (PPMS) at 300 K. All scale bars are 20 nm except for C, which is 10 nm.

Figure 1 illustrates how the Fe_3_O_4_/Gd_2_O_3_-DHCA (FGDA) nanocubes were synthesized in this work. During the first steps of the reaction, Fe_3_O_4_/Gd_2_O_3_-oleic acid (FGOA) nanocubes were produced by the thermal decomposition of the metal oleate mixtures. In this procedure, both the temperature and duration of the reaction play important roles in determining the nanocube size. The FGOA nanocubes were obtained by refluxing the metal oleate mixtures at 310 °C for 30 min. According to the TEM pictures (Figure 2a,d), the FGOA nanocubes were 7.44 ± 0.10 nm in one dimension and showed monodispersity. After the ligand-exchange treatment, the FGDA nanocubes became slightly smaller (about 6.33 ± 0.09 nm) but were still monodisperse (Figure 2b,e). The change in size might have happened due to changes in the surface when the FGOA nanocubes were converted into FGDA nanocubes. In order to verify the composition and distribution of elements in the FGDA nanocubes, energy-dispersive X-ray spectroscopy (EDS) was performed. The EDS spectrum shows that iron, gadolinium and oxygen were the main elements present in the nanocubes. No other impurity elements can be detected except carbon element, which contributes from carbon film on copper mesh used in the EDS experiments. EDS mapping indicates that iron and gadolinium distribute uniformly across the FGDA nanocubes (Figure 2f,g). In addition, the high-resolution transmission electron microscopy (HRTEM) image (Figure 2c, inset in red) shows that the interplanar spacing within the nanocubes is 0.296 ± 0.02 nm, which corresponds to the (220) crystal planes in Fe_3_O_4_ [29].

In order to further analyze the FGDA nanocube composition, XRD analysis was performed. According to Figure 2h, the major diffraction peaks were consistent with the diffraction peaks of pure Fe_3_O_4_ and Gd_2_O_3_ [23,30], confirming that the FGDA nanocubes were constituted by uniformly distributed Fe_3_O_4_/Gd_2_O_3_ composites.

FGOA nanocubes are hydrophobic since they are covered by oleic acid, which is enriched in alkyl groups. In order to increase the hydrophilicity of FGOA nanocubes so they could be used as a contrast agent in vivo, FGOA was exchanged with DHCA via the ligand-exchange method (Figure 2i). Fourier-transform infrared spectroscopy (FTIR) was conducted to verify the nanocube surface modifiers before and after DHCA exchange. By comparing the FTIR spectra of FGOA and FGDA nanocubes (Figure 2i), it is possible to conclude that the two samples display distinct characteristic absorption peaks. On the one hand, the FGOA nanocubes have characteristic absorption peaks at 2937 and 2857 cm^−1^, corresponding to the stretching vibration of –CH_3_ and –CH_2_, respectively, which are both present in oleic acid. On the other hand, the FGDA nanocubes have the characteristic absorption peak at 1617 cm^−1^, corresponding to the benzene ring stretching vibration, which indicates that DHCA successfully modified the FGDA nanocubes.

In order to verify the magnetic properties of FGDA nanocubes, the field-dependent magnetization (*M*–*H*) curves (Figure 2j) were obtained from the physical property measurement system (PPMS) operating at 300 K and the Fe_3_O_4_ nanocubes were used as control. Figure 2j shows that both Fe_3_O_4_ and FGDA nanocubes were superparamagnetic and the magnetic saturation was reached at 0.2132 and 0.7612 emu·g^−1^, respectively. Even though both values were low (probably due to the small size of the nanocubes [31–32]), the FGDA nanocubes presented a higher magnetic saturation than their Fe_3_O_4_ counterparts, likely due to the addition of gadolinium.

### Relaxation rate measurement

**Figure 3:**
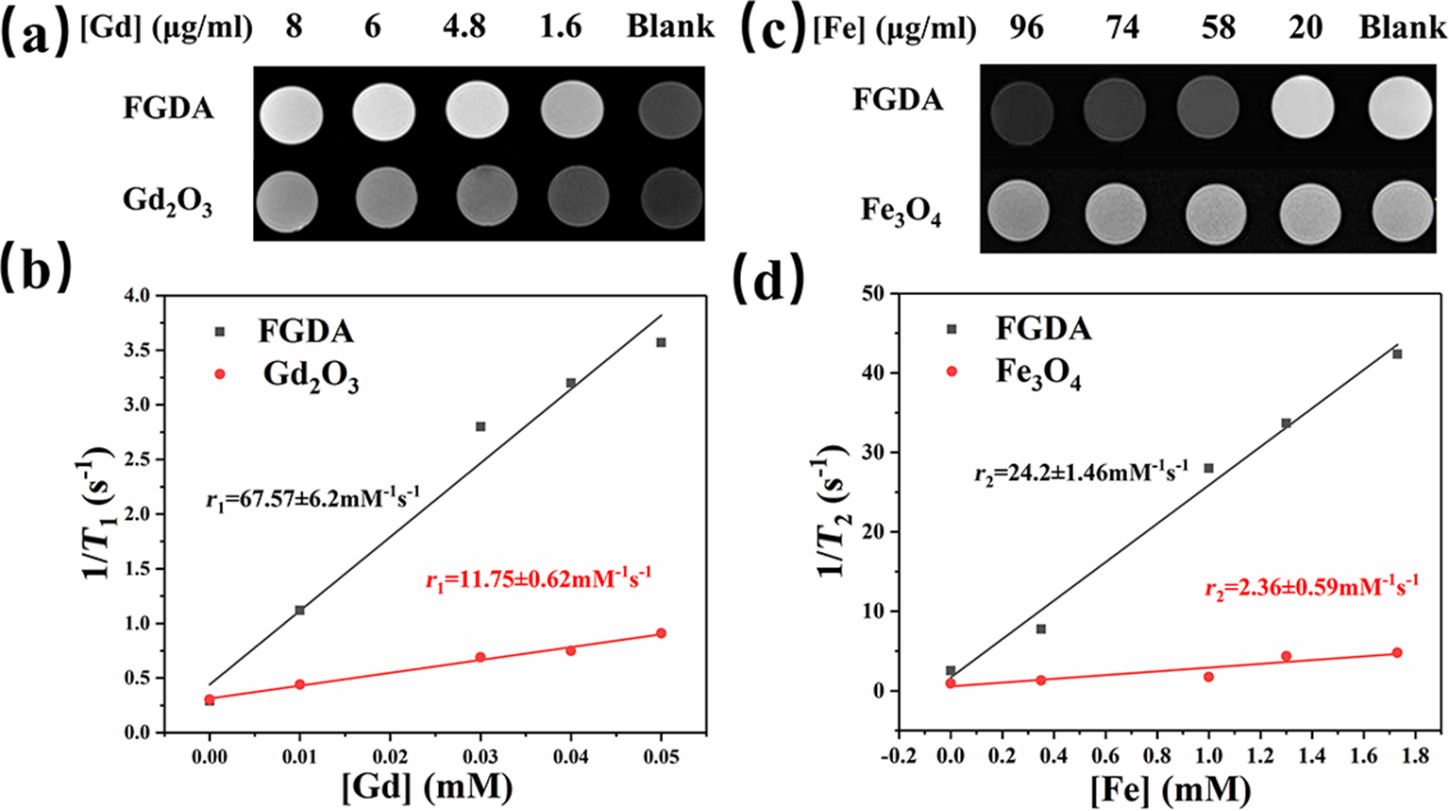
(a) *T*_1_-weigted MRI of FGDA nanocubes (top panel) and Gd_2_O_3_ nanoparticles (bottom panel). (b) Longitudinal relaxation rate, *r*_1_, calculated for FGDA nanocubes (black line) and Gd_2_O_3_ nanoparticles (red line). (c) *T*_2_-weigted MRI imaging of FGDA (top panel) and Fe_3_O_4_ nanocubes (bottom panel). (d) Transverse relaxation rate, *r*_2_, of FGDA nanocubes (black line) and Fe_3_O_4_ nanocubes (red line).

Given that FGDA nanocubes are *T*_1_–*T*_2_ dual-mode contrast agents they should enhance MRI contrast. In order to verify this, different concentrations of FGDA nanocubes were submitted to MRI testing in which *r*_1_ and *r*_2_ were obtained. Fe_3_O_4_ nanocubes and Gd_2_O_3_ nanoparticles were scanned as control groups. As expected, FGDA nanocubes provided a better contrast when compared to either Gd_2_O_3_ nanoparticles or Fe_3_O_4_ nanocubes (Figure 3a,c). To further investigate the degree to which FGDA nanocubes increased the MRI contrast, *r*_1_ and *r*_2_ values were calculated and found to be 67.57 ± 6.2 and 24.2 ± 1.46 mM^−1^·s^−1^, respectively. Both values were higher than those obtained for the control groups (*r*_1_ = 11.75 ± 0.62 mM^−1^·s^−1^ for Gd_2_O_3_ nanoparticles and *r*_2_ = 2.36 ± 0.59 mM^−1^·s^−1^ for pure Fe_3_O_4_ nanocubes). This value of *r*_1_ was also higher than previously reported [20], probably due to the special structure of the FGDA nanocubes. Taken together, these results confirm that Gd_2_O_3_- or Fe_3_O_4_-containing FGDA nanocubes significantly increase the MRI contrast when compared to pure Gd_2_O_3_ nanoparticles or to Fe_3_O_4_ nanocubes and therefore can be used as a sensitive *T*_1_–*T*_2_ dual-mode contrast agent in MRI.

### Cytotoxicity of FGDA nanocubes

**Figure 4:**
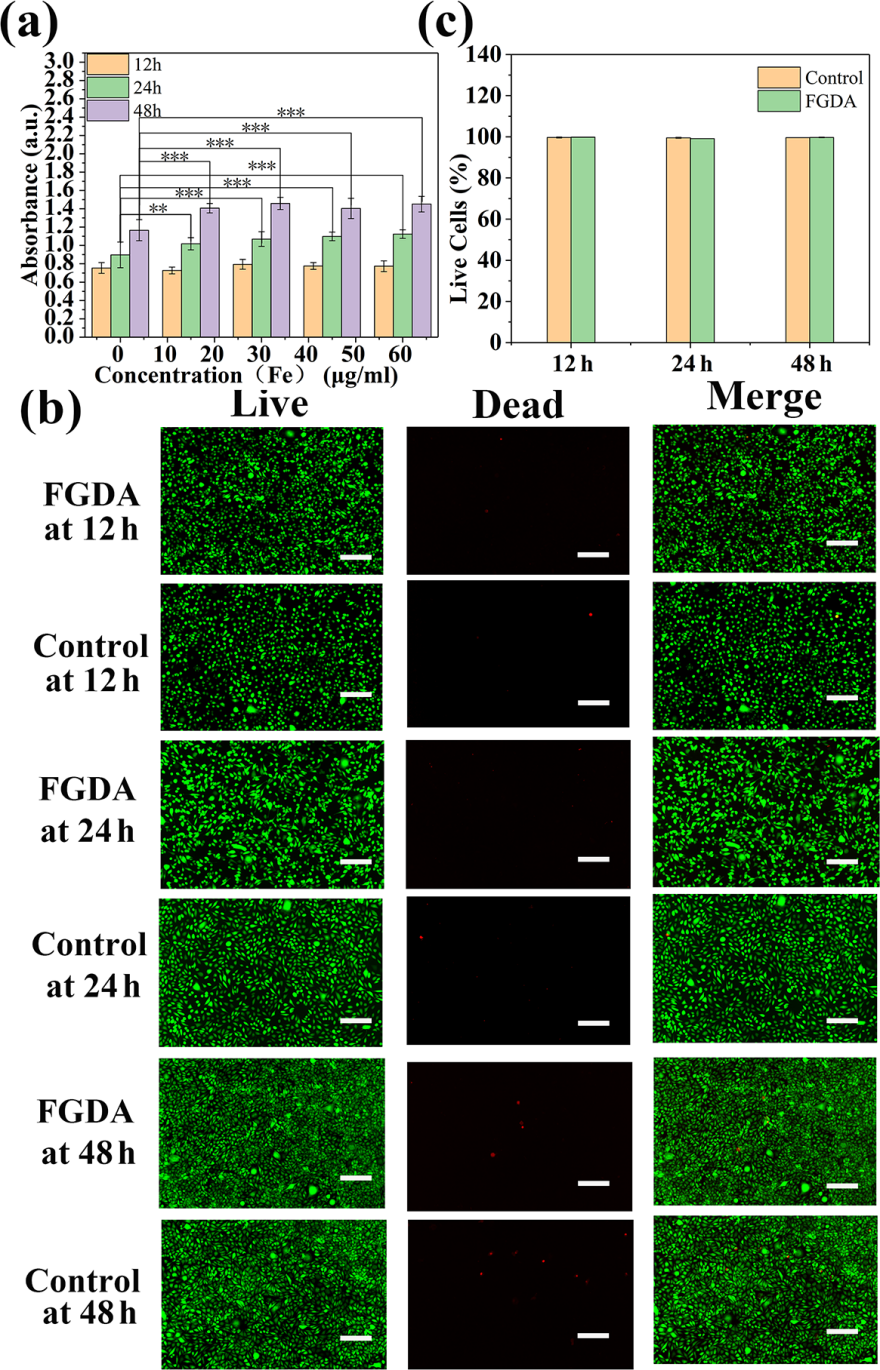
(a) CCK-8 cell viability test of L929 cells incubated with FGDA at different Fe concentrations for 12, 24, and 48 h. (b) Live–dead staining of control and FGDA-treated groups (Fe concentration: 60 μg/mL) after 12, 24, and 48 h of incubation. (c) Quantification of live cells shown in (b). Scale bar: 100 μm. ** and *** represent *P* < 0.01 and *P* < 0.001, respectively.

In order to use the FGDA nanocubes as a contrast agent for in vivo MRI it is imperative to first test their cytotoxicity in vitro [33]. In this work, the CCK-8 assay was performed to measure the viability of L929 cells upon exposure to FGDA nanocubes. Figure 4a indicates that at 12 h after treatment there were no differences in cell viability between the control and groups treated with different concentrations of FGDA nanocubes. At that stage, the L929 cells were probably still adapting to the media containing FGDA nanocubes and therefore were not proliferating. At later time points (24 and 48 h), the L929 cells treated with the FGDA nanocubes were proliferating normally and their proliferation rate was slightly higher than that measured for the control groups (*P* < 0.01).

In order to further test the cytotoxicity of FGDA nanocubes, live–dead staining was performed. The results showed that there was no difference in cellular morphology or viability between the control and treated groups (Figure 4b,c). In conclusion, FGDA nanocubes have no negative impact on cell viability or proliferation, which suggests that these nanocubes can be used for in vivo applications.

### In vivo MRI with FGDA nanocubes as a contrast agent

**Figure 5:**
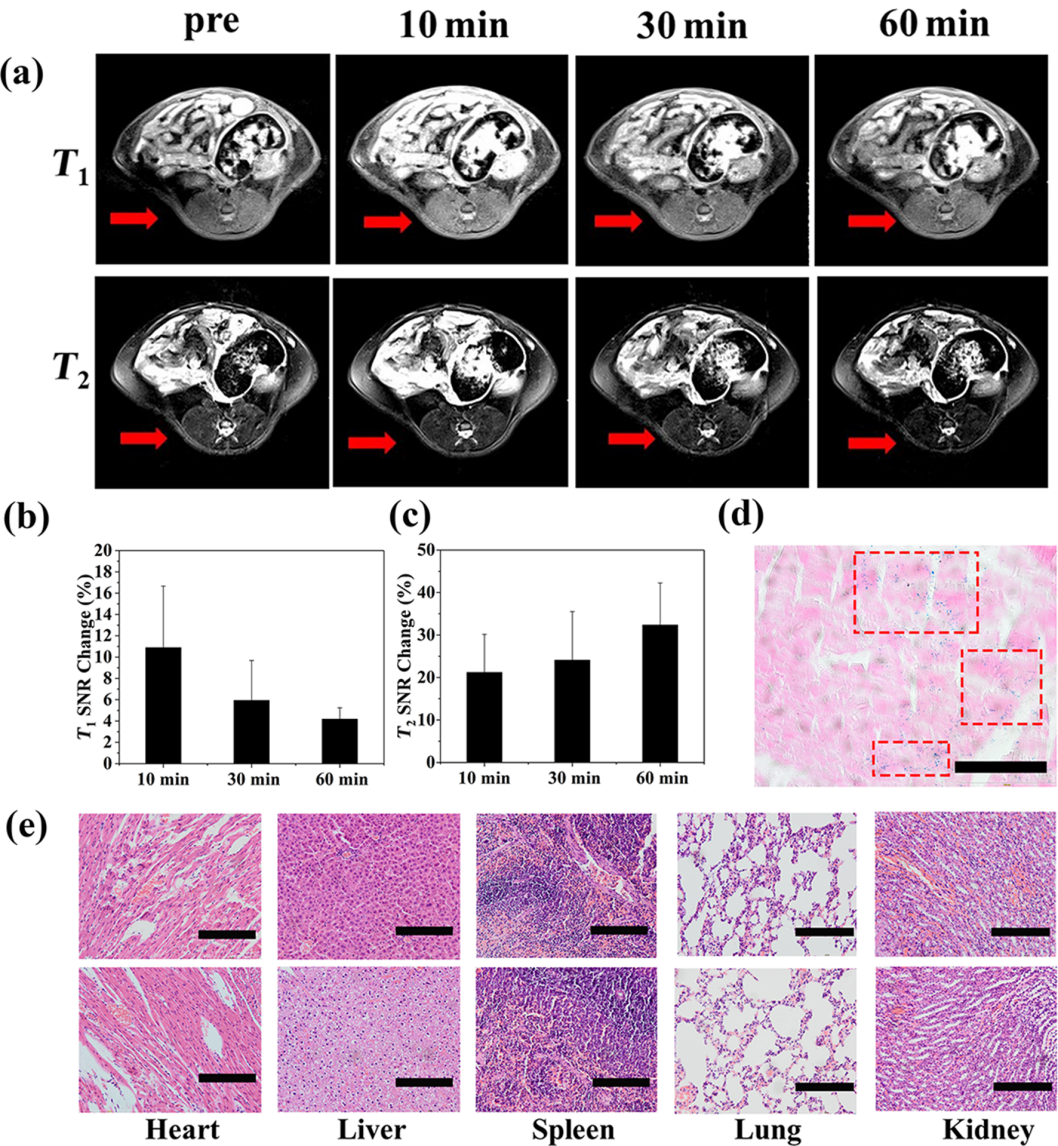
(a) T_1_WI and T_2_WI in vivo images of SD rats before and after intravenous injection of FGDA nanocubes. (b) *T*_1_ and (c) *T*_2_ SNR change post-intravenous injection of FGDA nanocubes. (d) Prussian blue staining in the lumbar muscle of a rat that received an intravenous injection of FGDA nanocubes. Scale bar: 100 μm. (e) H&E staining of heart, liver, spleen, lung and kidney. Top images: control group, bottom images: rats that were sacrificed two weeks after receiving an injection of FGDA nanocubes. Scale bar: 200 μm.

To visualize the contrast and image quality provided by the FGDA nanocubes in vivo, *T*_1_-weighted imaging (T_1_WI) and *T*_2_-weighted imaging (T_2_WI) were performed in Sprague Dawley (SD) rats. After intravenous injection of a 0.8 mg Fe/kg dose, FGDA nanocubes spread systemically through the bloodstream. The lumbar muscle was chosen as the MRI region of interest and the signal-to-noise ratio (ΔSNR) was measured at several time points. As shown in Figure 5a (top) and Figure 5b, T_1_WI was significantly enhanced 10 minutes after the FGDA nanocube injection (ΔSNR = 10.9%) when compared to the preinjection images. However, ΔSNR decreased consistently 30 min (ΔSNR = 5.94%) and 60 min (ΔSNR = 4.18%) after injection when compared with the 10 min time point. This result indicates that T_1_WI FGDA nanocubes have a short-term effect in terms of increasing contrast ΔSNR during MRI in vivo. On the other hand, the effects of FGDA nanocube injection on T_2_WI were prolonged, as shown in Figure 5a (bottom) and Figure 5c. Ten minutes after injection, ΔSNR was 21.21% and it increased to 24.08% and 32.34%, 30 and 60 min post injection, respectively. The discrepancy between T_1_WI and T_2_WI is probably due to the low gadolinium and high iron concentrations in FGDA nanocubes. When the nanocubes were gradually excreted, Fe_3_O_4_ maintained an oversaturated state and kept generating the *T*_2_ signals since its concentration was higher than Gd_2_O_3_ in the nanocubes. Prussian blue staining further confirmed the presence of iron in lumbar muscles, indicating that the *T*_1_ and *T*_2_ signal changes were indeed induced by the FGDA nanocubes (Figure 5d). Taken together these results show that the FGDA nanocubes can be used as a *T*_1_–*T*_2_ dual-mode contrast agent for in vivo MRI.

### Toxicity of FGDA nanocubes in vivo

Two weeks after the rats received the intravenous injection containing 2 mg Fe/kg FGDA nanocubes, their organs were harvested, fixed and processed for hematoxylin and eosin (H&E) staining for a qualitative evaluation of the in vivo toxicity. Figure 5e demonstrated that the FGDA nanocubes did not cause significant side effects in major organs when compared with the control group. This result reinforces that FGDA nanocubes are safe to be used as a contrast agent for in vivo MRI.

## Conclusion

Uniformly distributed FGDA nanocubes were synthesized via the thermal decomposition method. FGDA nanocubes had high *r*_1_ and *r*_2_ values which enhanced the MRI quality. The in vitro and in vivo toxicity assays demonstrated that the FGDA nanocubes did not cause significant side effects in cells or animals. In vivo T_1_WI and T_2_WI images showed that FGDA nanocubes can be used as a *T*_1_–*T*_2_ dual-mode contrast agent in MRI. Collectively, the results show that FGDA nanocubes may serve as a potential MRI tracker in research and clinical settings. In the future, the insertion of special modifiers on the FGDA nanocubes may increase their applicability in the clinical settings.

## Experimental

### Materials

Oleic acid (C_18_H_34_O_2_, OA) and iron(III) chloride (FeCl_3_) were purchased from Tianjin Beichenfangzheng Chemical Reagent Factory. Hexane (C_6_H_14_), absolute ethanol (C_2_H_6_O), sodium hydroxide (NaOH) and tetrahydrofuran (C_4_H_8_O, THF) were purchased from Tianjin Kaitong Chemical Reagent Co, Ltd. Oleylamine (C_18_H_37_N), 1-octadecene (C_18_H_36_), sodium oleate (C_18_H_33_NaO_2_), 3,4-dihydroxyhydrocinnamic acid (C_9_H_10_O_4_, DHCA), gadolinium acetate hydrate (Gd(CH_3_CO_2_)_3_·*x*H_2_O) and gadolinium chloride hexahydrate (GdCl_3_·6H_2_O) were purchased from Rhawn technology Co, Ltd. Ultrapure water was purified by Ulupure (UPR-II, China). All the reagents used in the experiments were of analytical grade and used as received without further purification.

### Synthesis of metal oleate precursor

The methods used for synthesizing metal oleate precursors were described in detail in a previous study [34]. 10 mmol sodium oleate was dissolved in a solution containing 60 mL ultrapure water and 20 mL ethanol, 5.0 mmol iron(III) chloride and 1.0 mmol gadolinium chloride hexahydrate were dissolved in 20 mL ultrapure water in a beaker. Then, the mixture was added to a 250 mL three-necked flask dropwise where it was heated and refluxed at 75 °C for 4 h. The reaction was cooled to room temperature and 20 mL hexane was added afterwards. Next, the mixture was transferred to a separating funnel where the organic phase, on top, was collected in a beaker and the aqueous phase, at the bottom, was discarded. The collected organic phase was washed with water in a separating funnel. The obtained metal oleate complex was dried at 55 °C for 24 h to form a ceraceous product. The ferric oleate was synthesized in a similar way.

### Synthesis of Fe_3_O_4_/Gd_2_O_3_-OA (FGOA) nanocubes

The uniformly distributed FGOA nanocubes were synthesized by the one-step thermal decomposition method. 1.12 g of metal oleate precursor, 0.17 mL of oleic acid and 15 mL of 1-octadecene were added into a 250 mL three-necked flask. The reaction system was heated up to 200 °C for 30 min, then heated up again to 310 °C at a rate of 4 °C/min and then submitted to refluxing for 30 min. All procedures mentioned above were performed under nitrogen atmosphere. When the reaction mixture was cooled down to room temperature, 80 mL of ethanol was added to the mixture to precipitate the nanocubes. Then, the hydrophobic nanocubes were collected by centrifugation at 8000 rpm for 5 min, washed several times using ethanol and hexane and the final product, the FGOA nanocubes, was resuspended in 3 mL of hexane.

### Synthesis of Fe_3_O_4_-OA nanocubes

0.92 g of ferric oleate, 0.17 mL of oleic acid and 15 mL of 1-octadecene were added to a 250 mL three-necked flask. The reaction system underwent the same heating and refluxing steps described in the previous section (also under nitrogen atmosphere). When the reaction mixture was cooled down to room temperature, 80 mL of ethanol was added to precipitate the nanocubes. Next, the hydrophobic nanocubes were collected by centrifugation at 8000 rpm for 5 min, washed several times using ethanol and hexane and the final product, the Fe_3_O_4_-OA nanocubes, was resuspended in 3 mL of hexane.

### Synthesis of Gd_2_O_3_-OA nanoparticles

0.334 g of gadolinium acetate hexahydrate, 4 mL of OA, 6 mL of oleylamine and 10 mL of 1-octadecene were added to a 250 mL three-necked flask. In order to remove volatile impurities, the reaction mixture was heated up to 100 °C for 1 h under nitrogen atmosphere. Then, it was heated up again to 310 °C (4 °C/min) and submitted to refluxing for 30 min. When the reaction mixture was cooled down to room temperature, 80 mL of ethanol was added to precipitate the nanoparticles. The final product, the Gd_2_O_3_-OA nanoparticles, was collected by centrifugation at 5000 rpm for 3 min, washed in ethanol and hexane and resuspended in 3 mL of hexane.

### Synthesis of hydrophilic Fe_3_O_4_/Gd_2_O_3_-DHCA (FGDA) nanocubes

Briefly, 200 mg of DHCA and 60 mL of THF were added to a 250 mL three-necked flask. 100 mg of FGOA nanocubes and 20 mL of THF were added separately to a beaker. Then, the solution in the beaker was added to the three-necked flask dropwise. The reaction mixture was heated up to 50 °C and submitted to refluxing for 4 h. All the experimental steps performed in the three-necked flask were under nitrogen atmosphere. When the obtained mixture was cooled down to room temperature, 5 mL of NaOH was added to the mixture to form a precipitate. The final products were collected by centrifugation at 10000 rpm for 10 min. Finally, the hydrophilic FGDA nanocubes were redispersed in 3 mL of ultrapure water. The water soluble Fe_3_O_4_-DHCA nanocubes and the Gd_2_O_3_-DHCA nanoparticles were synthesized using similar methods.

### Characterization of nanocubes and nanoparticles

A high-resolution transmission electron microscope (HRTEM, JEM-2010F, Japan), operated at an acceleration voltage of 200 kV, was used to investigate the morphology and size of the nanocubes. Energy-dispersive X-ray spectroscopy (EDS, Oxford, X-Max^N^, UK) was used to analyze the distribution of the elements within the samples. The samples were ultrasonically homogenized for 30 minutes and an 8 μL aliquot was collected in a copper mesh and kept at 55 °C for 2 h. After drying, the samples were placed in the HRTEM for imaging.

X-ray diffraction (XRD, UltimalV, Japan; Cu Kα (λ = 1.5406 Å)) was used to determine the crystalline structure of the nanocubes. The diffractometer was operated at 40 kV and 30 mA. Scanning was performed at 2θ values ranging from 20 to 80° at a rate of 0.05°·s^−1^.

Fourier-transform infrared spectroscopy was performed on the samples over a wavenumber range of 4000–400 cm^−1^ using the reflection mode (Bruker Alpha, Germany).

The field-dependent magnetization (*M*–*H*) curves were obtained from a physical property measurement system (PPMS) (Quantum Design, California, USA) operating at 300 K. Inductively coupled plasma atomic emission spectroscopy (ICP-AES, iCAP7400, Thermo Scientific, USA) was used to test the Fe and Gd concentrations in the samples. For this procedure, 100 μL of nanocubes were dissolved in 500 μL of concentrated nitric acid and then heated up to 200 °C until the liquid evaporated. This procedure was repeated several times until the solution was clear and transparent. After that, the solution was diluted in 10 mL of ultrapure water and finally could be used for testing.

In vitro and in vivo magnetic resonance images were captured by a 3.0 T MRI scanner (MAGNETOM Skyra, Siemens Healthcare, Erlangen, Germany).

The longitudinal (*r*_1_) and transverse (*r*_2_) sample relaxation time rates were measured to evaluate the properties of the dual-mode contrast agents. Fe_3_O_4_-DHCA and Gd_2_O_3_-DHCA were used as controls at different concentrations: 0, 20, 58, 74, and 96 μg/mL and 0, 1.6, 4.8, 6, and 8 μg/mL, respectively.

The nanoparticles were diluted in normal saline and their relaxation time rates were measured using the 3.0 T magnetic resonance scanner with a 15-channel knee coil with *T*_1_-weighted imaging (TR/TE = 1000/11 ms) and *T*_2_-weighted imaging (TR/TE = 4000/70 ms).

### Cytotoxicity assay

L929 cells were seeded onto a 96-well plate at a density of 1 × 10^4^ cells per well, cultured in 200 μL Dulbecco’s Modified Eagle’s Medium (DMEM, Gibco, Grand Island, USA) supplemented with 10% fetal bovine serum (FBS, Gibco, USA) and 1% antibiotic/antimycotic solution (Thermo Fisher, USA) and incubated at 37 °C with 5% CO_2_ for 24 h. Then, the culture medium was discarded and fresh media was added containing 15, 30, 45, and 60 μg/mL FGDA nanocubes, respectively. A cell counting kit (CCK-8) was used to evaluate the nanocube cytotoxicity at 12, 24, and 48 h. The absorbance was detected using a microplate reader (Biorad iMark, USA) at a wavelength of 450 nm. Each group had six samples in parallel.

Live–dead staining was also performed to observe FGDA nanocube cytotoxicity. The staining was performed using Calcein AM (C1430, Thermo Fisher, USA) and ethidium homodimer 1 (E1169, Thermo Fisher, USA). Briefly, L929 cells were seeded onto a 24-well plate at a density of 3 × 10^4^ cells per well. After 24 h of incubation, the media was replaced by 1 mL of fresh cell media containing 60 μg/mL FGDA nanocubes. The live–dead staining was performed after the cells were incubated with FGDA nanocubes for 12, 24, and 48 h. Images of the stained cells were collected with an inverted phase-contrast microscope (Nikon, TiS, Japan).

### In vivo magnetic resonance imaging

All the animal experiments were performed in compliance with the guidelines for animal experimentation from the Shanxi Medical University. MRI was performed in three Sprague Dawley (SD) rats that received FGDA nanocubes as the contrast agent. The animals were anesthetized with 2% isoflurane inhalation anesthesia while the MRI was being performed. T_1_WI and T_2_WI were acquired by the turbo spin echo (TSE) sequence with TR/TE = 550/14 ms and TR/TE = 2510/101 ms, respectively. After the FGDA nanocubes were injected in the tail vein, at a dose of 0.8 mg Fe/kg, the MRI was performed at 10, 30 and 60 min post-injection.

### Histological staining

Immediately after the MRI, the rats were euthanized by a lethal intravenous injection of chloral hydrate. The lumbar muscles were harvested, placed into 4% paraformaldehyde solution and processed for Prussian blue staining.

### In vivo toxicity evaluation of FGDA nanocubes

FGDA nanocubes were injected intravenously at a dose of 2 mg Fe/kg. Alternatively, the control group received a normal saline intravenous injection. After two weeks, the rats were euthanized by overdose with an intravenous injection of chloral hydrate. Their main organs (heart, liver, spleen, lung and kidney) were harvested, placed into 4% paraformaldehyde and processed for hematoxylin and eosin (H&E) staining.

### Data analysis

The data were expressed as mean ± standard deviation. Statistical significance was assessed using the Student’s *t*-test and the values were considered significant at *P* < 0.05. “*”, “**”, and “***” represent *P* < 0.05, *P* < 0.01, and *P* < 0.001, respectively.
